# Nomogram for Postoperative Permanent Neurologic Dysfunction Following Acute Type A Aortic Dissection Surgery: A Multicentral Retrospective Study

**DOI:** 10.1002/clc.70518

**Published:** 2026-11-05

**Authors:** Jian Chen, Jianan Li, Juncheng Yu, Meijuan Dai, Chiyin Wang, Junming Zhu, Jue Wang

**Affiliations:** ^1^ Department of Cardiac Surgery The First Affiliated Hospital of Wenzhou Medical University Wenzhou Zhejiang China; ^2^ Department of Cardiovascular Surgery, Beijing Anzhen Hospital Capital Medical University Beijing China

**Keywords:** aortic dissection, neurologic dysfunction, nomogram, prediction model, risk factors, supra‐aortic branch vessels dissection

## Abstract

**Background:**

Permanent neurologic dysfunction (PND) often complicates following surgery for acute type A aortic dissection (ATAAD) and is strongly correlated with unfavorable outcomes. Consequently, developing an effective diagnostic method to identify high‐risk patients is of considerable importance.

**Methods:**

This retrospective study, covering 2022 to 2024 across two centers, ultimately included 117 ATAAD patients with Supra‐Aortic Branch Vessel (SABV) dissection from our center (2022–2023) as the training set and 52 ATAAD patients with SABV dissection from a secondary center (2024) as the validation set. We used logistic regression to identify predictors and developed a nomogram, which was assessed using the area under the ROC curve, calibration and decision curve analysis. External validation was conducted using the validation set.

**Results:**

In this study, 169 cases were finally collected (117 in the training set and 52 in the validation set), of whom 64 patients developed postoperative PND. Four risk factors were identified: brachiocephalic trunk dissection at the bifurcation plane, chronic kidney disease, age over 50, and cross‐clamp duration over 168 min. The AUC was 0.809 for the training set and 0.736 for the validation set. The nomogram model demonstrated acceptable predictive performance, and decision curve analysis potential clinical utility for predicting postoperative PND risk for thresholds of 1%–90% in the training set and 3%–75% in the validation set.

**Conclusions:**

This study developed and validated a predictive model for assessing the risk of postoperative PND in patients undergoing ATAAD surgery, which may assist clinicians in individualized risk assessment.

## Graphical abstract

Legend: SABV: Supra‐Aortic Branch Vessel; PND: permanent neurologic dysfunction; CKD: Chronic kidney disease; BCT: brachiocephalic trunk; LCCA: left common carotid artery.

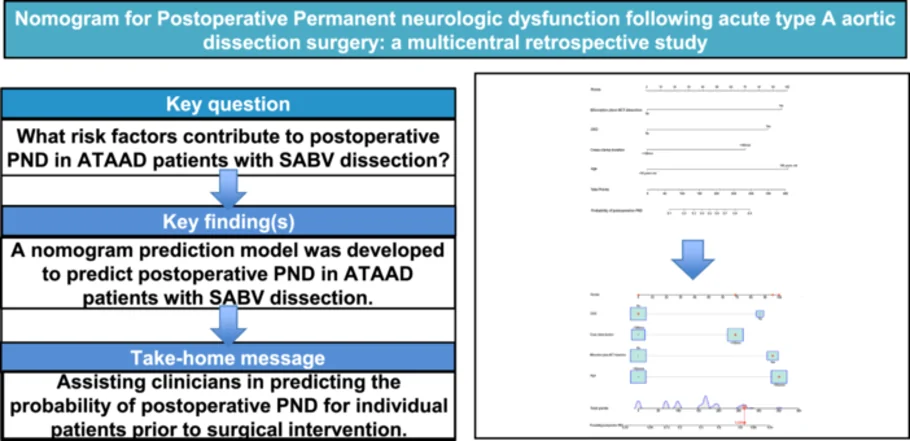

**Abbreviations**
AF: atrial fibrillation
ATAAD: acute type A aortic dissection
AUC: area under the curve
BCT: brachiocephalic trunk
CABG: coronary artery bypass grafting
CAD: coronary artery disease
CKD: Chronic kidney disease
CM: cerebral mal‐perfusion
CPB: cardiopulmonary bypass
CRP: C‐reactive protein
CT: computed tomography
CTA: computed tomography angiography
DCA: decision curve analysis
GFR: glomerular filtration rate
HCA: hypothermic circulatory arrest
LCCA: left common carotid artery
LSCA: left subclavian artery
MRI: magnetic resonance imaging
OR: odds ratio
PND: permanent neurologic dysfunction
RCCA: right common carotid artery
ROC: Receiver operating characteristic
RSCA: right subclavian artery
SABV: supra‐aortic branch vessels
TND: temporary neurologic dysfunction

## 1 Introduction

Acute type A aortic dissection (ATAAD) represents a cardiovascular condition characterized by high mortality rates and numerous postoperative complications. Advances in imaging technology and surgical techniques have contributed to a reduction in mortality rates [1]. However, ATAAD accompanied by end‐organ mal‐perfusion remains associated with substantial mortality. In particular, neurologic dysfunction arising from cerebral mal‐perfusion (CM) has been identified as an indicator of poor prognosis [2].

Postoperative neurologic complications encompass both temporary neurologic dysfunction (TND) and permanent neurologic dysfunction (PND). The occurrence of postoperative PND is associated with prolonged hospitalization, increased treatment costs, and diminished quality of life [3]. Therefore, postoperative PND represents a significant concern following acute type A aortic dissection (ATAAD) surgery.

Cerebral mal‐perfusion has been identified as a risk factor for postoperative neurologic complications in many studies [4, 5, 6]. And considering that supra‐aortic branch vessels (SABV) are related to cerebral blood flow supply [2, 7, 8, 9, 10]. Consequently, this study concentrated on examining the correlation between aortic dissection involving the aortic arch and its branches and the incidence of postoperative PND. In doing so, we aimed to develop an extensive clinical prediction model that is primarily based on imaging findings.

Finally, Our study used multicenter data to construct and validate a predictive model for predicting the probability of postoperative PND. We believed that this model would enable clinicians to quickly assess the probability of a patient developing postoperative PND, so that early prevention and intervention can be carried out.

## 2 Materials and Methods

### 2.1 Patients

This retrospective study encompassed patients diagnosed with acute type A aortic dissection (ATAAD) who received surgical intervention at two medical institutions between January 2022 and December 2024. The ethics committees of both Beijing Anzhen Hospital and the First Affiliated Hospital of Wenzhou Medical University granted approval for this retrospective study. In accordance with ethical guidelines for retrospective studies, individual patient informed consent was not required. The diagnoses of ATAAD and SABV dissection were established based on preoperative aortic computed tomography angiography (CTA). Furthermore, postoperative PND was identified through postoperative cranial magnetic resonance imaging (MRI) or computed tomography (CT) examination. All surgical procedures were conducted following standard protocols by experienced surgical teams at the two departments. For the analysis, patients with a history of stroke prior to surgery were excluded, and only ATAAD patients with SABV dissection were included in the final study cohort.

### 2.2 Surgical Technique

There were individual differences in the operation plans of patients with ATAAD, which were decided by cardiac surgeons in our hospital according to the situation during the operation. The following is the routine procedure. All the patients were operated under general anesthesia with cryogenic cardiopulmonary bypass. After successful anesthesia, the patient was placed in a supine position, and the right axillary artery was set free for backup. The median sternum was opened to free the ascending aorta, aortic arch, brachiocephalic trunk (BCT), left common carotid artery (LCCA), and left subclavian artery (LSCA). The right axillary artery was cannulated, and the right atrial was cannulated to establish cardiopulmonary bypass. The ascending aorta was blocked through the opening of the proximal brachial trunk of the ascending aorta, the ascending aorta was cut longitudinally, and cold‐blooded cardioplegic fluid was injected into the coronary opening to arrest the heart. The root of the aorta and the opening of the coronary artery were investigated, and Bentall or simple ascending aorta replacement was performed at the proximal end according to the situation. When the body temperature dropped to 25°C, the brachiocephalic trunk, left common carotid artery, and left subclavian artery were blocked, and selective antegrade cerebral perfusion (ACP) was established through the axillary artery. Cut off the diseased aortic arch, select a suitable descending aortic stent, insert it into the true lumen of the descending aorta, then continuously suture it to the edge of the descending aorta and the distal end of the four‐branched artificial blood vessel. Clamping three branches of the four‐branched artificial blood vessel, the remaining branch was connected to the extracorporeal circulation arterial tube to restore blood flow to the lower limbs. Gradually re‐warming, the four‐branched artificial blood vessel was anastomosed to the left common carotid artery, left subclavian artery, and the brachiocephalic trunk, respectively. Finally, the proximal end of the artificial blood vessel was anastomosed to the proximal ascending aorta. Release the aortic cross‐clamp and wait for the heart to beat. Continue to adjust the extracorporeal circulation until the circulation is stable. Check all anastomotic sites for bleeding, close the chest layer by layer, and transfer the patient to the Cardiovascular Care Unit (CCU). Because the cannulation strategy, ACP technique, and target hypothermic level were largely standardized in our centers, these variables were not included as major candidate predictors in the present analysis.

### 2.3 Definitions

We defined the starting plane as the plane where the Supra‐aortic branch vessels diverge from the aortic arch. The bifurcation plane was defined as the plane where the brachiocephalic trunk divides into the right common carotid artery (RCCA) and the right subclavian artery (RSCA). The two planes we defined were illustrated in Figure S1. Subsequently, We define permanent neurological dysfunction (PND) as the emergence of a new postoperative hemorrhagic or ischemic stroke, confirmed by cranial CT or MRI, where the lesion remains unresolved upon follow‐up assessment within 6 months postoperatively, or the development of coma. Additionally, in line with the inclusion and exclusion criteria of other relevant articles, we excluded patients with preoperative PND. The study flow chart is presented in Figure S2.

### 2.4 CTA Acquisition and Image Analysis

Preoperative CTA was performed using a multidetector CT scanner (64‐slice CT; LightSpeed VCT, GE Healthcare, Milwaukee, WI, USA) with a slice thickness of 0.625 mm according to the institutional standard arterial‐phase contrast‐enhanced protocol. CTA images were analyzed on a dedicated three‐dimensional workstation, and multiplanar reconstructions perpendicular to the aortic centerline were generated to ensure accurate image interpretation and short‐axis measurements.

Imaging analysis focused on the involvement of supra‐aortic branch vessels at the starting plane and bifurcation plane, as well as lumen‐related measurements when applicable. Given the retrospective design of this study, implementation of a strict double‐blind protocol was inherently not feasible. Nevertheless, measures were taken to ensure the highest possible degree of blinding during image evaluation and to reduce potential observer bias. Specifically, all CTA images were independently reviewed by two experienced observers with expertise in cardiovascular imaging, both of whom were blinded to the patients' clinical characteristics and postoperative neurologic outcomes. Any discrepancies were resolved by consensus. In cases of disagreement, the final assessment was reached by consensus. Interobserver agreement was assessed according to the type of imaging variable: the intraclass correlation coefficient (ICC) was used for continuous measurements, and Cohen's kappa coefficient was used for categorical imaging assessments.

### 2.5 Data Collection and Potential Predictors

In this study, we systematically collected patients' data through an exhaustive review of their hospital records, ancillary examinations, and laboratory results. The collected information was categorized into three distinct types: imaging data, clinically relevant data, and laboratory examination data. The imaging data encompassed preoperative involvement of the supra‐aortic branch vessels (SABV) at both the starting and bifurcation planes, as well as the extent of involvement. The laboratory examination data included preoperative levels of lactate, C‐reactive protein (CRP), cardiac troponin T (cTnT), and D‐dimer. The clinically relevant data comprised demographic and lifestyle factors such as age, gender, tobacco and alcohol use, as well as medical history including hypertension, diabetes mellitus (DM), coronary artery disease (CAD), atrial fibrillation (AF), chronic kidney disease (CKD), Marfan's syndrome, coronary artery tears, preoperative shock, and previous dissection. Additionally, intraoperative variables such as the methods of operation, cardiopulmonary bypass (CPB) duration, cross‐clamp duration, and duration of hypothermic arrest were included.

### 2.6 Logistic Regression Analysis and Development of a Predictive Model

A total of 169 ATAAD patients with SABV dissection underwent surgery from the two centers were divided into training and validation sets at a ratio of approximately 7:3, consistent with the approach taken in most articles [11, 12]. A Univariable logistic regression analysis was first performed in the training cohort to identify potential predictors of postoperative PND. Subsequently, a multivariable logistic regression identified risk factors for postoperative PND in ATAAD patients with SABV dissection. In the primary analysis, continuous variables retained as candidate predictors were dichotomized based on optimal cut‐off values derived from receiver operating characteristic (ROC) analysis, thereby enhancing the clinical interpretability and applicability of the model. Model complexity was additionally evaluated using the events‐per‐variable (EPV) ratio to assess the potential risk of overfitting. A nomogram was then created and validated. To evaluate the potential impact of dichotomizing continuous predictors, a sensitivity analysis was conducted using the same four predictors as in the primary model, with age and cross‐clamp duration entered as continuous variables, whereas CKD and BCT dissection at the bifurcation plane remained binary variables.

### 2.7 Performance of the Nomogram

The model's predictive accuracy was assessed using discrimination and calibration. ROC curves estimated the nomogram's discriminatory ability, with an AUC over 0.80 indicating good discrimination [13]. Additionally, Calibration of the nomogram, accompanied by the Hosmer–Lemeshow test, was used to determine consistency between predicted and observed postoperative PND occurrence. Internal validation in the training cohort was performed using bootstrap resampling with 1000 repetitions. For external validation, the model developed in the training cohort was directly applied to the independent validation cohort without refitting. Calibration in the external validation cohort was further evaluated using the calibration curve, calibration intercept, and calibration slope. Finally, a decision curve analysis (DCA) was performed to evaluate the clinical practicability of the nomograms [14].

### 2.8 Statistical Analysis

Statistical analysis was conducted using SPSS27 and R v.4.3.2. Categorical variables were shown as frequencies (percentages), normal continuous variables as means ± standard deviations, and skewed continuous variables as medians (interquartile range). Categorical data were analyzed with χ2 or Fisher's exact tests, normal continuous data with *t*‐tests or ANOVA, and skewed data with Mann–Whitney *U* tests. Logistic regression and nomogram were created in R using the ‘rms' package. All *p*‐values were two‐tailed, with significance set at *p* < 0.05.

## 3 Results

### 3.1 Patients' Characteristics

First, we compared the baseline characteristics of all 166 ATAAD patients from our center, including patients with and without SABV dissection, as shown in Table 1. Notably, we found a significant difference in the incidence of preoperative SABV dissection between the two groups (84.6% vs 64.0%, *p* = 0.007). Therefore, our subsequent research will focus on analyzing patients with ATAAD and SABV dissection.

**TABLE 1:** Baseline characteristics between the PND and non‐PND groups of all patients from our center.

	Overall (*n* = 166)	PND (*n* = 52)	Non‐PND (*n* = 114)	*p* value
Preoperative parameters				
Demographic (%)				
Age (years)	49.9 ± 12.5	54.3 ± 11.0	48.0 ± 12.7	0.002**
Male (gender)	128 (77.1)	40 (76.9)	88 (77.2)	0.969
Tobacco use (%)	63 (38.0)	18 (34.6)	45 (39.5)	0.550
Alcohol use (%)	46 (27.7)	15 (28.8)	31 (27.2)	0.825
Medical history				
hypertension (%)	111 (66.9)	34 (65.4)	77 (67.5)	0.784
DM (%)	8 (4.8)	3 (5.8)	5 (4.4)	0.707
CAD (%)	9 (5.4)	3 (5.8)	6 (5.3)	1.000
AF (%)	10 (6.0)	5 (9.6)	5 (4.4)	0.289
CKD (%)	38 (22.9)	18 (34.6)	20 (17.5)	0.018*
Marfan syndrome (%)	14 (8.4)	4 (7.7)	10 (8.8)	1.000
Coronary artery tears (%)	83 (50.0)	29 (55.8)	54 (47.4)	0.315
Preoperative shock (%)	4 (2.4)	1 (1.9)	3 (2.6)	1.000
Previous dissection (%)	2 (1.2)	2 (3.8)	0 (0)	0.097
SABV dissection (%)	117 (70.5)	44 (84.6)	73 (64.0)	0.007**
Laboratory results				
Lactic acid (mmol/L)	2.1 (1.3,3.2)	2.5 (1.6,4.3)	1.9 (1.3,3.0)	0.020*
CRP (mg/L)	5.1 (1.7,20.4)	2.6 (1.4,11.1)	5.5 (1.9,22.8)	0.103
cTnT (μg/L)	19.3 (8.0,84.7)	37.0 (14.0,132.5)	17.6 (6.9,60.3)	0.009**
D‐Dimer (mg/L)	9.6 (2.8,20.0)	17.0 (4.3,20.0)	7.7 (1.9,20.0)	0.025*
Intraoperative parameters				
Operative methods				
Bentall (%)	65 (39.2)	19 (36.5)	46 (40.4)	0.641
Total‐arch replacement (%)	121 (72.9)	44 (84.6)	77 (67.5)	0.022*
Concomitant CABG (%)	84 (50.6)	22 (42.3)	62 (54.4)	0.149
CPB duration (min)	259.0 (214.0,317.0)	258.0 (218.0,358.0)	259.0 (213.0,315.0)	0.316
Cross‐clamp duration (min)	191.8 ± 64.6	200.2 ± 68.3	187.9 ± 62.7	0.258
HCA time (min)	22 (18,25)	21.0 (16.0,24.5)	22.0 (19.0,26.0)	0.224

*Note*: *p < 0.05, **p < 0.01.

Abbreviations: AF, atrial fibrillation; CABG, coronary artery bypass grafting; CAD, coronary artery disease; CKD, chronic kidney disease; CPB, cardiopulmonary bypass; CRP, C‐reactive protein; DM, diabetes mellitus; HCA, hypothermic circulatory arrest; PND, permanent neurologic dysfunction; SABV, supra‐aortic branch vessels.

As depicted in Table 2, 117 patients with SABV dissection were assigned to the training set, among them, 44 (37.6%) patients developed postoperative PND after ATAAD surgery.

**TABLE 2:** Baseline characteristics between the postoperative PND and non‐PND groups in training set.

	Overall (*n* = 117)	PND (*n* = 44)	Non‐PND (*n* = 73)	*p* value
Preoperative parameters				
Demographic (%)				
Age (years)	50.0 ± 11.9	54.4 ± 10.1	47.3 ± 12.2	0.001**
Male (gender)	94 (80.3)	36 (81.8)	58 (79.5)	0.814
Tobacco use (%)	47 (40.2)	17 (38.6)	30 (41.1)	0.793
Alcohol use (%)	37 (31.6)	14 (31.8)	23 (31.5)	1.000
Medical history				
hypertension (%)	79 (67.5)	30 (68.2)	49 (67.1)	0.906
DM (%)	6 (5.1)	3 (6.8)	3 (4.1)	0.671
CAD (%)	7 (6.0)	3 (6.8)	4 (5.5)	1.000
AF (%)	6 (5.1)	4 (9.1)	2 (2.7)	0.196
CKD (%)	21 (17.9)	13 (29.5)	8 (11.0)	0.014*
Marfan syndrome (%)	8 (6.8)	2 (4.5)	6 (8.2)	0.708
Coronary artery tears (%)	62 (53.0)	25 (56.8)	37 (50.7)	0.520
Preoperative shock (%)	1 (0.9)	1 (2.3)	0 (0)	0.376
Previous dissection (%)	2 (1.7)	2 (4.5)	0 (0)	0.139
Image characteristics				
Starting plane dissection				
BCT (%)	100 (85.5)	40 (90.9)	60 (82.2)	0.195
True lumen smaller than false lumen (%)	53 (45.3)	24 (54.5)	29 (39.7)	0.119
LCCA (%)	67 (57.3)	32 (72.7)	35 (47.9)	0.009**
True lumen smaller than false lumen (%)	22 (18.8)	11 (25.0)	11 (15.1)	0.224
LSCA (%)	70 (59.8)	27 (61.4)	43 (58.9)	0.793
True lumen smaller than false lumen (%)	20 (17.1)	8 (18.2)	12 (16.4)	0.805
Bifurcation plane dissection				
BCT (%)	39 (33.3)	22 (50.0)	17 (23.3)	0.004**
True lumen smaller than false lumen (%)	21 (17.9)	9 (20.5)	12 (16.4)	0.624
LCCA (%)	21 (17.9)	11 (25.0)	10 (13.7)	0.141
True lumen smaller than false lumen (%)	6 (5.1)	4 (9.1)	2 (2.7)	0.196
LSCA (%)	26 (22.2)	11 (25.0)	15 (20.5)	0.648
True lumen smaller than false lumen (%)	8 (6.8)	2 (4.5)	6 (8.2)	0.708
RCCA (%)	27 (23.1)	17 (38.6)	10 (13.7)	0.003**
RSCA (%)	6 (5.1)	4 (9.1)	2 (2.7)	0.196
Laboratory results				
Lactic acid (mmol/L)	2.3 (1.3,3.2)	2.3 (1.4,3.7)	2.2 (1.3,3.1)	0.300
CRP (mg/L)	5.1 (1.6,20.6)	3.4 (1.6,11.9)	5.1 (1.6,28.4)	0.358
cTnT (μg/L)	22.2 (7.4,94.0)	32.7 (9.4,125.8)	18.6 (5.8,84.9)	0.057
D‐Dimer (mg/L)	9.9 (2.9,20)	18.1 (5.1,20.0)	7.5 (2.0,16.8)	0.009**
Intraoperative parameters				
Operative methods				
Bentall (%)	53 (45.3)	16 (36.4)	37 (50.7)	0.132
Total‐arch replacement (%)	108 (92.3)	41 (93.2)	67 (91.8)	0.783
Concomitant CABG (%)	38 (32.5)	15 (34.1)	23 (31.5)	0.840
CPB duration (min)	253 (213,331)	261 (217,365.5)	239 (202.5,318)	0.130
Cross‐clamp duration (min)	187.3 ± 68.9	205.8 ± 70.6	176.2 ± 65.8	0.023*
HCA time (min)	22 (16,25)	20.5 (15.5,25)	22 (16,25)	0.767

*Note*: *p < 0.05, **p < 0.01.

Abbreviations: AF, atrial fibrillation; BCT, brachiocephalic trunk; CABG, coronary artery bypass grafting; CAD, coronary artery disease; CKD, chronic kidney disease; CPB, cardiopulmonary bypass; CRP, C‐reactive protein; DM, diabetes mellitus; HCA, hypothermic circulatory arrest; LCCA, left common carotid artery; LSCA, left subclavian artery; PND, permanent neurologic dysfunction; RCCA, right common carotid artery; RSCA, right subclavian artery.

Table 2 presents the baseline characteristics of patients with SABV dissection, divided into Non‐PND and PND groups. The average patient age was 50, with 80.3% male and 19.7% female. CKD was present in 17.9% of patients, and hypertension in 67.5%. Patients with postoperative PND were older (*p* = 0.001) and more likely to have CKD (29.5% vs. 11.0%, *p* = 0.014). The PND group also showed higher preoperative D‐dimer levels (*p* = 0.009), more frequent LCCA dissection at the starting plane (72.7% vs. 47.9%, *p* = 0.009), higher BCT dissection at the bifurcation plane (50.0% vs. 23.3%, *p* = 0.004), increased RCCA dissection (38.6% vs. 13.7%, *p* = 0.003), and longer cross‐clamp duration (*p* = 0.023).

The interobserver reproducibility of CTA evaluations demonstrated a high level of consistency. The intraclass correlation coefficients (ICCs) for continuous measurements of true‐ and false‐lumen diameters ranged from 0.88 to 0.95, signifying good to excellent agreement. Cohen's kappa coefficients for categorical assessments of supra‐aortic branch vessel dissection varied from 0.82 to 0.92, indicating almost perfect agreement. Comprehensive results are provided in Table S1.

### 3.2 Selection of Predictive Factors

To enhance the practical applicability of the final clinical model, a Receiver Operating Characteristic (ROC) analysis was conducted for each continuous variable to ascertain the optimal cut‐off value. Subsequently, the continuous variables were converted into categorical variables based on these determined cut‐off values.

Given the multitude of variables involved, we employed a two‐step approach to filter the clinical features: univariable logistic regression identified potential predictors, and multivariable logistic regression further analyzed these predictors. The results, as presented in Table 3, indicate the following: age greater than 50 years (OR = 6.33, 95% CI: 2.37–16.90, *p* < 0.001), chronic kidney disease (CKD) (OR = 4.92, 95% CI: 1.56–15.52, *p* = 0.006), BCT dissection at bifurcation plane (OR = 5.82, 95% CI: 2.12–15.97, *p* = 0.001), and cross‐clamp duration exceeding 168 min (OR = 3.57, 95% CI: 1.32–9.65, *p* = 0.012). The final multivariable model included four predictors among 44 postoperative PND events, corresponding to an events‐per‐variable (EPV) ratio of 11.

**TABLE 3:** The prediction model with univariable and multivariable logistic regression.

Variables	Univariable analysis		Multivariable analysis	
	OR (95% CI)	*p*‐value	OR (95% CI)	*p*‐value
Age > 50 years	4.29 (1.90–9.67)	0.001**	6.33 (2.37–16.90)	< 0.001**
CKD	3.41 (1.28–9.08)	0.014*	4.92 (1.56–15.52)	0.006**
Preoperative D‐dimer > 17.5 mg/L	3.61 (1.62–8.06)	0.002**		
Preoperative cTnT > 8.2 μg/L	2.49 (1.01–6.14)	0.048*		
Starting plane LCCA dissection	2.9 (1.29–6.49)	0.010**		
Bifurcation plane BCT dissection	3.29 (1.48–7.34)	0.004**	5.82 (2.12–15.97)	0.001**
RCCA dissection	3.97 (1.61–9.77)	0.003**		
CPB duration > 373 min	5.07 (1.48–17.37)	0.010*		
Cross‐clamp duration > 168 min	2.32 (1.05–5.13)	0.038*	3.57 (1.32–9.65)	0.012*

*Note*: *p < 0.05, **p < 0.01.

As a sensitivity analysis, age and cross‐clamp duration were re‐entered into the multivariable logistic regression model as continuous variables, while CKD and BCT dissection at the bifurcation plane remained binary variables. The direction of associations remained consistent with the primary analysis. BCT dissection at the bifurcation plane (OR = 4.53, 95% CI: 1.79–11.50, *p* = 0.001), CKD (OR = 3.71, 95% CI: 1.22–11.31, *p* = 0.021), age (OR = 1.07 per 1‐year increase, 95% CI: 1.02–1.11, *p* = 0.002), and cross‐clamp duration (OR = 1.01 per 1‐min increase, 95% CI: 1.00–1.01, *p* = 0.038) remained significantly associated with postoperative PND (refer to Table S2). The AUCs of the continuous‐variable model were 0.779 in the training cohort and 0.620 in the external validation cohort, compared with 0.809 and 0.736, respectively, for the primary categorical model. These findings suggest that the associations of the identified predictors were generally consistent, although the discriminative ability of the continuous‐variable model was somewhat diminished relative to the primary categorical model, particularly within the external validation cohort.

### 3.3 Model Evaluation and Comparison

First, we categorized the predictors as either clinical or imaging variables. Clinical variables included CKD, Cross‐clamp duration exceeding 168 min and age greater than 50 years, while imaging variables included BCT dissection at the bifurcation plane. Then we constructed three models: one incorporating clinical variables, another incorporating imaging variables, and a third integrating both clinical and imaging variables. The performance of these models was assessed through Receiver Operating Characteristic (ROC) analysis, as depicted in Figure 1. As shown in Table 4, in the training cohort, the AUCs for the clinical, imaging and integrated models were 0.753 (95% CI: 0.665–0.841), 0.634 (95% CI: 0.544–0.723), and 0.809 (95% CI: 0.729–0.888). The corresponding sensitivities were 81.8%, 50.0%, and 54.5%, while the specificities were 54.8%, 76.7%, and 90.4%, respectively. The DeLong test revealed that the integrated model exhibited a significantly higher AUC compared to the clinical and imaging models, with all *p*‐values for these comparisons being less than 0.05. In the validation set, the integrated model also demonstrated superior performance in predicting the probability of postoperative PND, as shown in Table 4, with an AUC value of 0.736 (95% CI: 0.601–0.871). Consequently, the integrated model was selected as the final predictive model, and a nomogram was developed to visualize it.

**FIGURE 1:**
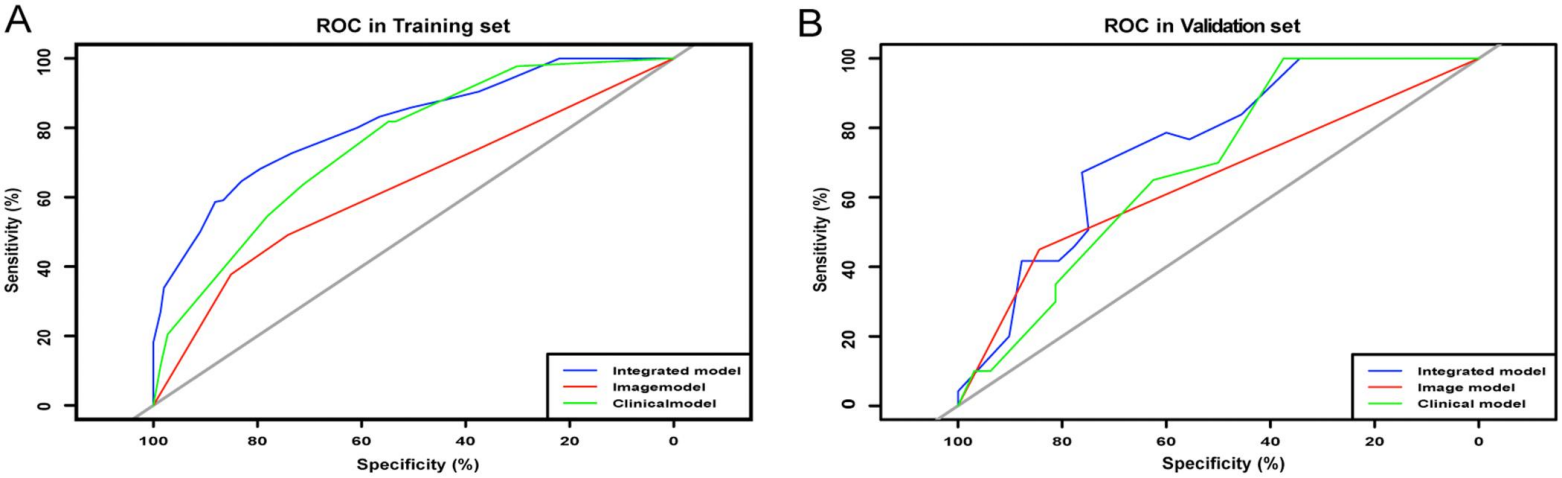
Comparison among three models by ROC in the training set and validation set. (A) The receiver operating curves of three models in training set. And the area under the ROC for discrimination of the integrated model in training set was 0.809 (95% CI: 0.729–0.888). (B) The receiver operating curves of three models in validation set. In the validation set, the area under the ROC for discrimination of the integrated model remained as high as 0.736 (95% CI: 0.601–0.871).

**TABLE 4:** AUCs of three models in training set and validation set.

Model	Training set			Validation set		
	AUC (95% CI)	Sensitivity	Specificity	AUC (95% CI)	Sensitivity	Specificity
Imaging model	0.634 (0.544–0.723)	0.500	0.767	0.647 (0.518–0.776)	0.450	0.844
Clinical model	0.753 (0.665–0.841)	0.818	0.548	0.689 (0.549–0.830)	1.000	0.375
Integrated model	0.809 (0.729–0.888)	0.545	0.904	0.736 (0.601–0.871)	0.650	0.750

Subsequently, a nomogram was established combining the key clinical characteristics described above. The nomogram shows the relative contribution of each factor to the risk of postoperative PND. According to the nomogram, age greater than 50 years was considered the strongest predictor of postoperative PND, followed by BCT dissection at bifurcation plane, CKD and cross‐clamp duration exceeding 168 min (Figure 2A).

**FIGURE 2:**
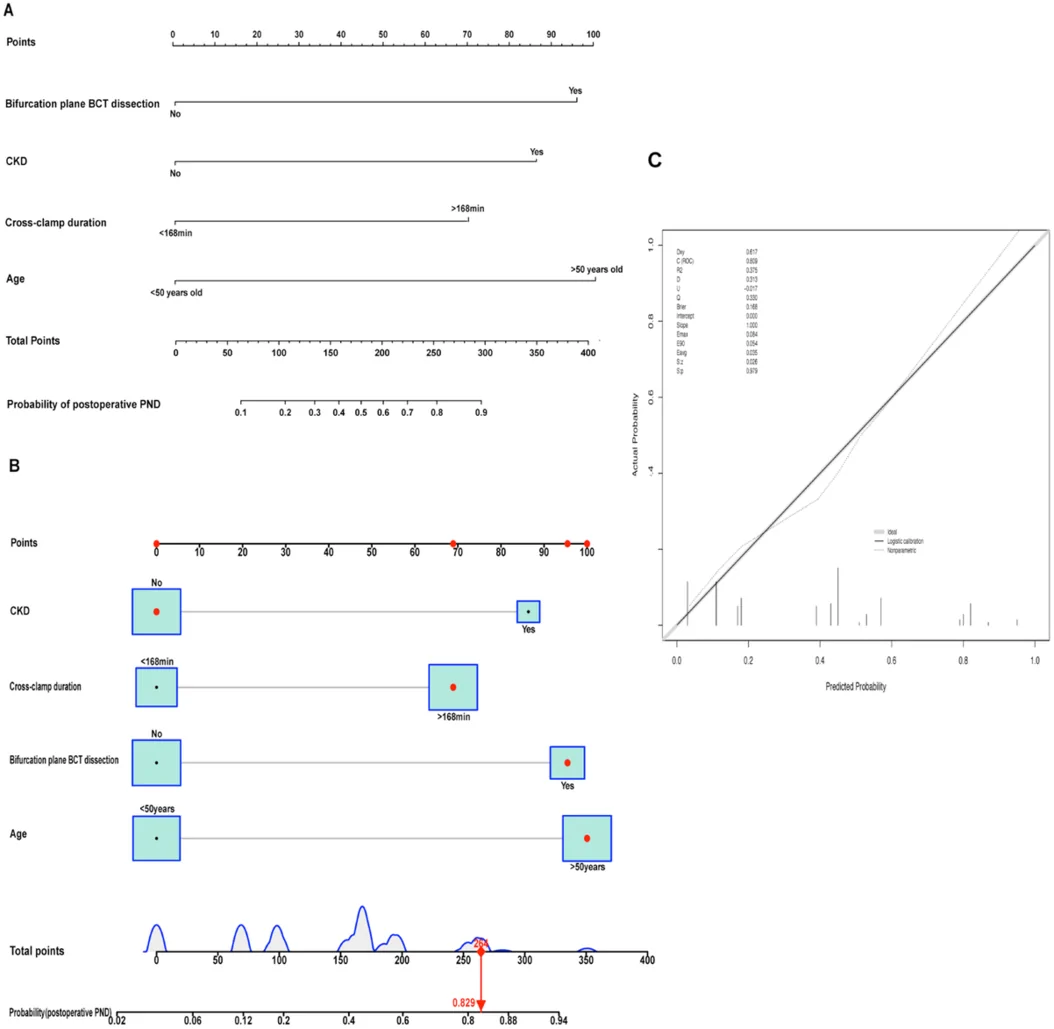
The nomogram for the integrated model. (A) The nomogram used for predicting the probability of postoperative PND include age greater than 50 years, preoperative CKD, BCT dissection at bifurcation plane and cross‐clamp duration exceeding 168 min. (B) The dynamic nomogram reveals the probability of postoperative PND in a patient after ATAAD surgery. The estimated probability of postoperative PND reached 0.829. (C) Calibration curves of the integrated model in the training set. *p*‐value > 0.05 in the Hosmer–Lemeshow test indicated an agreement between the predicted probabilities and observed outcomes.

The dynamic nomogram revealed the surgical outcomes in a patient with ATAAD surgery. This patient was greater than 50 years old, suffered from BCT dissection at bifurcation plane and experienced cross‐clamp duration exceeding 168 min. The estimated probability of postoperative PND for this case was 0.829. (Figure 2B).

Additionally, the calibration curve of the integrated model presented no difference between the predicted and actual probabilities of postoperative PND occurrence in the training set (*p* = 0.979) (Figure 2C). In the external validation cohort, the calibration assessment revealed a discrepancy between the predicted and observed probabilities, indicated by a calibration intercept of 0.124 and a calibration slope of 0.597. The associated calibration curve is depicted in Figure S3.

Furthermore, the result of DCA revealed that the nomogram provided a net clinical benefit for predicting postoperative PND. As is shown in Figure 3, the integrated model demonstrated a significantly higher net benefit compared to the other models across most threshold probabilities. Moreover, for the integrated model, these benefits were observed within a risk threshold probability range of 1%–90% in the training set (Figures 3A) and 3%–75% in the validation set (Figure 3B).

**FIGURE 3:**
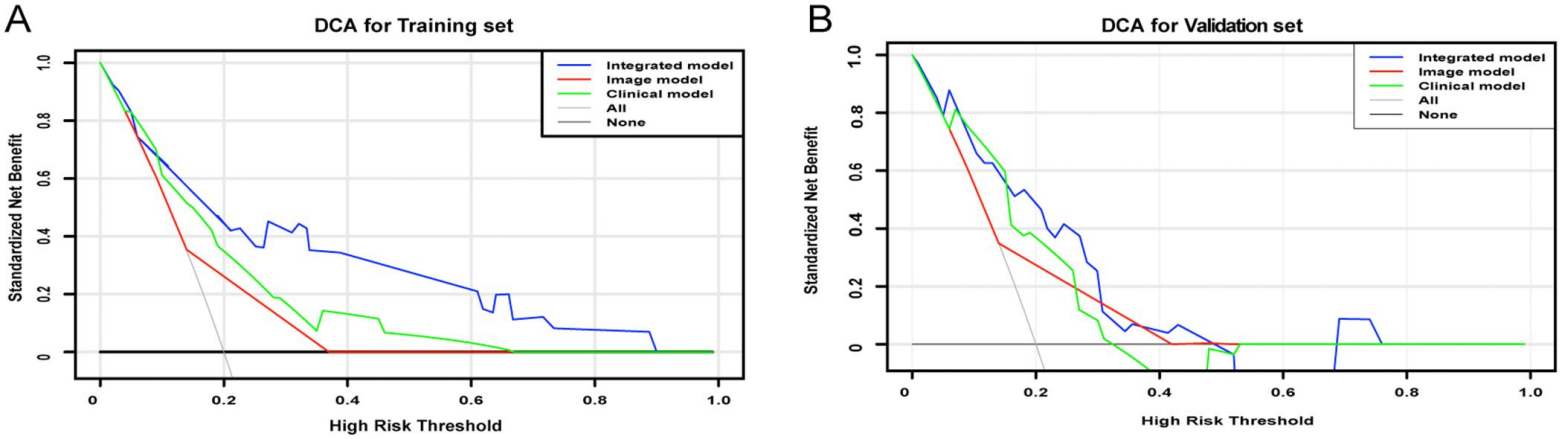
Comparison among three models by DCA in the training set and validation set. (A) The gray line assumes that all patients suffered from postoperative PND, while the black line represents the assumption that no patient experienced postoperative PND. Three different colors correspond to three models' decision curve analysis (DCA) in training set. And the blue line represents the integrated model's DCA in the training set. (B) The decision curve analysis of three models in validation set. And the blue line represents the integrated model's DCA in the validation set.

## 4 Discussion

Firstly, our analysis showed that patients with SABV dissection had a higher risk of postoperative PND. We used logistic regression on this cohort to identify risk factors and developed a nomogram‐based integrated model. The model's effectiveness and clinical benefit were assessed using ROC and DCA, and external validation demonstrated acceptable predictive performance in the independent validation cohort; however, the generalizability of the model requires further confirmation in larger and more diverse populations and its applicability should be interpreted within the context of ATAAD patients with SABV dissection.

In the present study, starting‐plane LCCA dissection was more frequent among patients who developed postoperative PND and was associated with postoperative PND in the univariable analysis. However, this variable was not retained as an independent predictor in the final multivariable model. Therefore, this finding should be interpreted as an unadjusted association rather than an independent predictive effect. Previous studies have suggested that carotid artery pathology may be associated with adverse neurologic outcomes [15, 16]. Given the important contribution of the LCCA to cerebral blood flow [17], its involvement may still be clinically relevant; however, its independent predictive value requires further investigation.

Furthermore, our findings showed a strong link between BCT dissection at the bifurcation plane and postoperative PND, similar to Cho et al.'s study [10]. However, they did not explore the correlation between the extent of involvement and PND incidence. According to previous research, because the BCT dissection at the bifurcation plane impacts both the blood flow of right CCA and SCA, which account for approximately 50% of the total cerebral blood supply [17]. This explained why BCT dissection at the bifurcation was closely tied to postoperative PND.

In addition, we found a close relationship between preoperative CKD and postoperative PND. A systematic review and meta‐analysis revealed that a 10 mL/min/1.73 m^2^ decrease in glomerular filtration rate (GFR) increases the risk of stroke by around 7% [18]. This finding was consistent with our research results that patients with CKD are at significantly greater risk of postoperative PND. Two potential mechanisms may explain this association. Firstly, the elevated levels of free radicals in CKD patients result in endothelial cell damage, thereby compromising the brain's capacity to regulate blood flow effectively [19]. Secondly, a prior study has suggested a genetic correlation between CKD and ischemic stroke, which may further elevate the likelihood of stroke occurrence in CKD patients [20].

Finally, we compared the integrated model with models based solely on clinically relevant data or imaging data alone. We found that the integrated model had higher sensitivity, specificity and clinical benefit, with predictors including imaging and clinically relevant variables. Some scholars have published a single‐center risk factor predictive model for postoperative TND after ATAAD surgery [21, 22]. In contrast, we used multicenter data to construct and validate a predictive model of postoperative PND in ATAAD patients with SABV dissection, which may provide preliminary evidence supporting its applicability in similar clinical settings.

From a clinical perspective, this model offers significant utility in two primary ways. Firstly, this model facilitates clinicians in the early evaluation of the probability that ATAAD patients with SABV dissection will develop postoperative PND, thereby enabling the prompt implementation of appropriate interventions. Secondly, this model will aid clinicians in providing personalized prediction of the probability of postoperative PND for each patient preceding operative interventions. By utilizing the above results, clinicians will be able to engage in thorough discussions with patients and their families about the risks associated with surgery, thereby effectively managing patient expectations concerning surgical outcomes.

### 4.1 Study Limitations

Regarding our study's limitations, several key points need highlighting. (I) The retrospective nature of the study inherently predisposes it to selection bias, information bias, and unmeasured confounding variables. (II) The sample size was relatively limited, particularly within the external validation cohort, and both participating centers were derived from relatively similar clinical settings, which may have affected the stability and generalizability of the predictive model. Despite the final model achieving an events‐per‐variable ratio of 11 and employing bootstrap resampling with 1000 iterations for internal validation, the potential for model optimism and overfitting cannot be entirely dismissed due to the limited number of postoperative PND events. This limitation may have led to an overestimation of the model's predictive performance. Consequently, further validation in larger and more diverse independent cohorts is recommended. (III) This study concentrated on examining the risk factors associated with postoperative PND in patients with ATAAD who exhibit SABV dissection. Consequently, our model was designed to predict the probability of postoperative PND in this specific patient population. (IV) Although the major cerebral protection strategy was relatively consistent in two centers, we did not perform a separate analysis of intraoperative technical variables such as subtle variations in perfusion management or surgeon‐specific operative preferences, which may still have influenced neurological outcomes.

## 5 Conclusions

In conclusion, our study indicates that advanced age, preoperative CKD, BCT dissection at bifurcation plane and prolonged cross‐clamp duration are independent risk factors for postoperative PND in ATAAD patients with SABV dissection. Furthermore, the nomogram we developed demonstrated an acceptable predictive capability for assessing the risk of postoperative PND in ATAAD patients with SABV dissection.

## Author Contributions

**Jian Chen:** writing – original draft, methodology, investigation. **Jianan Li:** writing – review and editing, data curation. **Juncheng Yu:** software. **Meijuan Dai:** data curation. **Chiyin Wang:** software. **Junming Zhu:** supervision. **Jue Wang :** supervision.

## Funding

The authors have nothing to report.

## Ethics Statement

This study was approved by the ethical review committee of First Affiliated Hospital of Wenzhou Medical University (KY2023‐R189‐01). The research ethics committee waived the requirement for informed consent for retrospective samples.

## Consent

In accordance with ethical guidelines for retrospective studies, individual patient informed consent was not required.

## Conflicts of Interest

The authors declare no conflicts of interest.

## Supporting information


**Figure S1:** Illustration of starting plane and bifurcation plane.

**Figure S2:** Flowchart of this study.

**Figure S3:** Calibration of the nomogram in the external validation cohort. The calibration curve assesses the concordance between predicted and observed probabilities of postoperative permanent neurologic dysfunction (PND) within the external validation cohort. The calibration intercept was determined to be 0.124, while the calibration slope was calculated as 0.597. The 45° reference line denotes ideal calibration.

Supporting File 1

Supporting File 2

## Data Availability

Data are available from the authors upon reasonable request and with permission of corresponding author (Jue Wang).
