# Supplementary material for: Nomogram for Postoperative Permanent Neurologic Dysfunction Following Acute Type A Aortic Dissection Surgery: A Multicentral Retrospective Study
Source: Clin Cardiol. 2026 Nov 5;49(11):e70518. doi: 10.1002/clc.70518 (PMC13739130; doi:10.1002/clc.70518)
Supplement: Supplementary file 1 [file CLC-49-e70518-s002.docx]

Supplementary Table1. Sensitivity analysis of the final multivariable logistic regression model with age and cross-clamp duration modeled as continuous variables.

| Variables | Adjusted OR (95%CI) | p-value |
| --- | --- | --- |
| Age (per 1-year increase) | 1.07(1.02-1.11) | 0.002** |
| CKD | 3.71(1.22-11.31) | 0.021* |
| Bifurcation plane BCT dissection | 4.53(1.79-11.50) | 0.001** |
| Cross-clamp duration (per 1-min increase) | 1.01(1.00-1.01) | 0.038* |

Note: *P<.05, **P<.01
