# Supplementary material for: Nomogram for Postoperative Permanent Neurologic Dysfunction Following Acute Type A Aortic Dissection Surgery: A Multicentral Retrospective Study
Source: Clin Cardiol. 2026 Nov 5;49(11):e70518. doi: 10.1002/clc.70518 (PMC13739130; doi:10.1002/clc.70518)
Supplement: Supplementary file 2 [file CLC-49-e70518-s004.docx]

**Supplementary Table S1. Interobserver reproducibility of CTA imaging assessments**

**Table S1A. Continuous imaging measurements**

| Parameter | ICC (95% CI) | Interpretation |
| --- | --- | --- |
| True lumen diameter of BCT  at the starting plane | 0.95 (0.92–0.97) | Excellent agreement |
| False lumen diameter of BCT  at the starting plane | 0.93 (0.88–0.95) | Excellent agreement |
| True lumen diameter of BCT  at the bifurcation plane | 0.94 (0.91–0.97) | Excellent agreement |
| False lumen diameter of BCT  at the bifurcation plane | 0.92 (0.87–0.95) | Excellent agreement |
| True lumen diameter of LCCA  at the starting plane | 0.94 (0.90–0.96) | Excellent agreement |
| False lumen diameter of LCCA  at the starting plane | 0.90 (0.85–0.94) | Excellent agreement |
| True lumen diameter of LCCA  at the bifurcation plane | 0.93 (0.89–0.96) | Excellent agreement |
| False lumen diameter of LCCA  at the bifurcation plane | 0.89 (0.84–0.93) | Good agreement |
| True lumen diameter of LSCA  at the starting plane | 0.92 (0.88–0.95) | Excellent agreement |
| False lumen diameter of LSCA  at the starting plane | 0.89 (0.82–0.93) | Good agreement |
| True lumen diameter of LSCA  at the bifurcation plane | 0.91 (0.86–0.95) | Excellent agreement |
| False lumen diameter of LSCA  at the bifurcation plane | 0.88 (0.82–0.92) | Good agreement |

**Table S1B. Categorical imaging measurements**

| Parameter | Cohen’s κ (95% CI) | Interpretation |
| --- | --- | --- |
| starting plane BCT dissection | 0.92 (0.85–0.99) | Almost perfect agreement |
| Bifurcation plane BCT dissection | 0.90 (0.82–0.98) | Almost perfect agreement |
| starting plane LCCA dissection | 0.88 (0.79–0.97) | Almost perfect agreement |
| Bifurcation plane LCCA dissection | 0.86 (0.77–0.95) | Almost perfect agreement |
| starting plane LSCA dissection | 0.84 (0.74–0.94) | Almost perfect agreement |
| Bifurcation plane LSCA dissection | 0.82 (0.71–0.93) | Almost perfect agreement |
| RCCA dissection | 0.89 (0.80–0.98) | Almost perfect agreement |
| RSCA dissection | 0.87 (0.77–0.97) | Almost perfect agreement |
